# Biological Activities of Various Extracts and Chemical Composition of *Trigonella monantha* C. A. Mey. subsp. *monantha* Grown in Iran 

**Published:** 2012

**Authors:** Akbar Esmaeili, Bahareh Rashidi, Shamsali Rezazadeh

**Affiliations:** a *Department of Chemical Engineering, North Tehran Branch, Islamic Azad University, Tehran, Iran. *; b *Iranian Academic Center for Education, Culture and Research, Institute of Medicinal Plant Research, Tehran, Iran. *

**Keywords:** Antioxidant activity, Essential oil, Total phenolics, Various extracts

## Abstract

The objective of this research was to study the biological activities of various extracts and the chemical composition of the essential oil *Trigonella monantha *C. A. Mey. subsp. monantha by gas chromatography (GC) and gas chromatography-mass spectroscopy (GC-MS), from the compounds derived from the aerial parts. The overall results of *T. monantha *tests allow us to conclude various extracts [hexane extract (HE), methanol extract (ME) and chloroform extract (CE)] of the test total phenolic, ABTS and DPPH while also testing *β*-carotene largest property antioxidant. The property antioxidant shows the extracts of both mechanisms of electron transfer and the hydrogen transfer it has gone through. The antimicrobial activity of the extracts of both samples was determined against seven Gram-positive and Gram-negative bacteria. The strongest activity exhibited by the *T. monantha *of ME was determined to be 49.58 μg/mL and was exhibited by ME.

## Introduction

*Trigonella *(Fabaceae), antiaromatic herbaceous plant is widely cultivated in Mediterranean countries and Asia (1). The literature records a variety of therapeutic actions of *T. monantha *including hypocholesterolaemia (2), hypoglycaemia (3), antibacterial (4), antiviral (5), anti-inflammatory activities (6), antioxidant and appetite stimulant (7), *etc. *Phytochemically, different parts of the plant contain several constituents such as alkaloids, proteins, flavonoids, saponins, *etc. *Several reports focus on trigonelline, a major active constituent having hypoglycemic activity, hypocholesterolemic, antiseptic, antimigraine, antitumor, mutagenic and osmoregular properties. (8-10). Free radicals were a major interest for early physicists and radiologists and much later were found to be a product of normal metabolism. Today, we well know that radicals cause molecular transformations and gene mutations in many types of organisms. Oxidative stress is well-known to cause many diseases (11), and scientists, in many different disciplines, have become more interested in natural sources which could provide active components to prevent or reduce its impact on cells (12, 13). Antioxidants can inhibit or delay the oxidation of an oxidizable substrate in a chain reaction and therefore, appear to be very important in the prevention of many diseases (13). The number of antioxidant compounds synthesized by plants as secondary products, mainly phenolics, serving in plant defence mechanisms to counteract reactive oxygen species (ROS) in order to survive, is currently estimated to be between 4000 and 6000 (14-17). The phenolic content and composition of plants and the products produced from them depend on genetic and environmental factors, as well as post-harvest processing conditions (18, 19). The antioxidant activities of phenolics are related to a number of different mechanisms, such as free radical-scavenging, hydrogen-donation, singlet oxygen quenching, metal ion-chelation, and acting as substrates for radicals such as superoxide and hydroxyl. A direct relationship has been found between the phenolic content and antioxidant capacity of plants. Antioxidants have been widely used as food additives to provide protection against the oxidative degradation of foods by free radicals. Since ancient times, spices added in different types of food to improve flavours, have been well known for their antioxidant capacities (20-22, 17). In this research, we studied antioxidants and antibacterial activities of various extract ME, HE and CE and chemical composition of the essential oil of *Trigonella monantha *C. A. Mey. Subsp. monantha by gas chromatography (GC) and gas chromatography-mass spectroscopy (GC-MS), from the compounds derived from the aerial parts.

## Experimental

*Plant material*

The aerial parts of *Trigonella *specie was collected during the flowering stage at the following places: *Trigonella monantha *C. A. Mey. subsp. monantha (Voucher No.7796), was collected in August 2010 from Karaj Province of Iran. Voucher specimens have been deposited at the Herbarium of the Research Institute of Forests and Rangelands (TARI), Tehran, Iran.

*Extraction of the oils*

Air-dried parts of *T. monantha *were separately subjected to hydrodistillation using a Clevenger type apparatus for 3 h. The essential oils were dried over anhydrous sodium sulphate and stored at 2°C in the dark before the analysis (40). *Isolation of the oil*

The seeds and aerial parts were mixed with hexane macerated and extracted to get the HE, then again extracted with chloroform and concentrated by means of vacuum evaporation in order to get CE. The remaining marc was then treated with methanol to get ME. The extracts were then subjected to preliminary phytochemical evaluation.

*Qualitative and quantitative analyses*

Most constituents were identified through the gas chromatography by the comparison of retention indices with either those of the literature or with those of authentic compound samples available in our laboratories. The retention indices were determined in relation to a homologous series of *n*-alkanes (C8-C28) under the same operating conditions. Further identification was made by the comparison of their mass spectra on both columns with either those stored in NIST 02 and Wiley 275 libraries or with mass spectra from the literature and our home made library. Component relative concentrations were calculated based on GC peak areas without using correction factors (30, 31).

*Preparations of the various extracts (ME, HE and CE)*

A portion of plant material (40 g) was successively extracted with 400 mL of ME, CE and HE (Merck, Darmstadt, Germany) by using a Soxhlet extractor (Isolab, Wertheim, Germany) for 72 h at a temperature not exceeding the boiling point of the solvent. The methanol extracts were filtered using Whatman filter paper (No: 1) and then concentrated in vacuum at 40°C using a Rotary Evaporator (Buchi, Flawil, Switzerland). The residue obtained was lyophilized in a Modulyo freeze-dryer (Edwards, Crawley, Sussex, UK) and the resulting powdered material was stored at -80°C until tested.

*Antimicrobial assay*

The antibacterial activity was evaluated by using the broth dilution method (32, 33). Nine bacteria species selected as representative of the class of Gram-positive or Gram-negative were tested: Gram-positive bacteria *Bacillus antracila *(PTCC 1274), *Bacillus cereus *(PTCC 1247), *Bacillus subtilis *(PTCC 1023)*, Staphylococcus epidermidis *(ATCC 12228), *Staphylococcus aureus *(ATCC 25923) and the Gram-negative bacteria *Escherichia coli *(ATCC 25922), *Pseudomonas *sp. (ATCC 85327), *Salmonella typhi *(ATCC 1231), and *Shikla fleksheneri *(ATCC 1042), were identified by the Research Center of Science and Industry, Tehran, Iran.

Microorganisms (obtained from enrichment culture of the microorganisms in 1 mL of Mueller-Hinton broth, incubated at 37°C for 12 h) were cultured on Mueller-Hinton agar medium. The following method was used to measure the antibacterial activity: 40 μL of diluted essential oil (40 μL oil in 2 mL DMSO (Dimethyl sulfoxide) 10%) was added to a 200 μL microbial suspension (1 loop from medium in physiological serum was compared with a 0.5 McFarland standard) in the 1st well of a microplate and 100 μL from this well was added to a 100 μL microbial suspension in the 2nd well, and this was continued until 8 wells in the microplate were filled. The microplate was then incubated at 37°C for 24 h (39).

*Antioxidant activity*

*Chemicals*

*β*-carotene, linoleic acid, DPPH (2,2-diphenyl-1-picryl hydrazyl), BHT were purchased from Sigma (Sigma-Aldrich GmbH, Steinheim, Germany). Pyrocatechol, Tween-20, FCR, sodium bicarbonate, ethanol, chloroform, methanol and the other chemicals and reagents were purchased from Merck (Darmstadt, Germany). All other unlabeled chemicals and reagents were of analytical grade.

*DPPH assay*

The hydrogen atom or electron donation ability of the corresponding extracts and some pure compounds was measured from the bleaching of purple coloured methanol solution of DPPH: This spectrophotometric assay uses the stable radical, DPPH as a reagent (34, 35). Fifty microliters of various concentrations of the extracts in methanol were added to 5 mL of a 0.004% methanol solution of DPPH. After a 30 min of incubation period at room temperature, the absorbance was read against a blank at 517 nm. The inhibition of free radical, DPPH, in percent (I%) was calculated in the following way:

I% = (Ablank - Asample / Ablank) × 100

Here, Ablank is the absorbance of the control reaction (containing all reagents except the test compound), and Asample is the absorbance of the test compound. Extract concentration providing 50% inhibition (IC50) was calculated form the graph plotted of inhibition percentage against the extract concentration. Tests were carried out in triplicate.

*β-Carotene-linoleic acid assay*

In this assay, antioxidant capacity is determined by measuring the inhibition of the volatile organic compounds and the conjugated diene hydroperoxides arising from linoleic acid oxidation (36). A stock solution of *β*-carotene/linoleic acid mixture was prepared as follows: 0.5 mg of *β*-carotene was dissolved in 1 mL of chloroform (HPLC grade); 25 μL of linoleic acid and Tween 40 were added. Next, chloroform was completely evaporated using a vacuum evaporator. Then, 100 mL of distilled water saturated with oxygen (30 min, 100 mL/min), was added with vigorous shaking. This reaction mixture (2500 μL) was dispensed to test tubes and 350 μL portions of the extracts prepared at 2 g/L concentrations were added and the emulsion system was incubated for up to 48 h at room temperature. The same procedure was repeated with the synthetic antioxidant, butylated hydroxytoluene (BHT) as positive control and a blank. After this incubation period, the absorbance of the mixtures was measured at 490 nm. Anti-oxidative capacities of the extracts were compared with those of BHT and blank.

*Determination of total phenolic compounds*

Total phenolic constituent in methanol extract of *T. monantha *were determined by literature methods involving FCR and gallic acid as standard (37). Extract solution (0.1 mL) containing extract was taken in a volumetric flask; 46 mL of distilled water and 1 mL FCR were added and the flask was thoroughly shaken. After 3 min, 3 mL of a solution of 7% Na2CO3 was added and the mixture was allowed to stand for 2 h with intermittent shaking. The absorbance was measured at 765 nm. The same procedure was repeated for all standard gallic acid solutions (0-1000 mg 0.1 mL-1) and a standard curve was obtained with the equation given below:

Absorbance = (0.0012 × Gallic acid μg) +0.0033 

involving FCR and gallic acid (both Sigma-Aldrich) as standard. Briefly, an aliquot (0.1 mL) of extract solution containing 1 mg of extract was transferred to a volumetric flask, 46 mL distilled water and 1 mL FCR was added and the flask was shaken thoroughly. After 3 min, 3 mL of solution (7% Na2CO3) was added and the mixture was allowed to stand for 2 h with intermittent shaking. The absorbance was measured at 765 nm. The same procedure was repeated for all standard gallic acid solutions (0.1 mL) and a standard curve was obtained according to the equation.

*ABTS radical cation scavenging assay*

The assay was performed by a slightly modified protocol. ABTS [(2,2’-azino-bis(3-ethylbenzothiazoline-6-sulphonic acid))] solution (7 mM) was reacted with ammonium persulfate (2.45 mM) solution and kept in the dark for 12-16 h to produce a dark coloured solution containing ABTS radical cations. The initial absorbance was measured at 734 nm. This stock solution was diluted with ethanol to give a final absorbance value of about 0.7 ± 0.02 (37) and equilibrated at 30°C. Different concentrations of the sample (50-250 g/mL) were prepared by dissolving the extracts in water. About 0.3 mL of the sample was mixed with 3 mL of ABTS working standard in a microcuvette. The decrease in absorbance was measured exactly 1 min after mixing the solution, then up to 6 min. The final absorbance was noted. The percentage of inhibition was calculated according to the formula:

%Inhibition = [(Acontrol × Asample) / Acontrol] × 100%

*Determination of flavonoid contents*

Total flavonoid content was assayed using aluminium chloride colorimetric assay employed the method of (38). The plant extracts were added in 2 mL HCl in a round bottom flask and were refluxed at 100°C for 30 min. The hydrophilic extract will be made up to 5 mL by using distilled water. To the mixture, 0.3 mL of 5% (w/v) NaNO2 and 3 mL of 10% AlCl3 was added. At the sixth min, 2 mL of 1 M NaOH was added to the mixture. The mixture was mixed well and the absorbance at 510 nm was read using the spectrophotometer. Total flavonoid content was expressed as mg catechin CE/g dry weight of sample. The experiment was replicated three independent assays.

## Results and discussion

*Essential oils analysis*

The percentage composition of the oils is given in Table 1; it is evident that the aerial parts of *T. monantha *oils are different mostly quantitatively than qualitatively.

**Table 1:** Chemical composition (%) of the *T. monantha *oil

**Compound**	a**RI**	b**%**	**IM**	**Compound**	a**RI**	b**%**	**IM**
**Hexanal**	819	9.5	RI,MS	Decanal	1204	5.5	RI,MS
**2-Heptanone**	892	0.8	RI,MS	Acetic acid, octyl ester	1211	1.0	RI,MS
**3,5-Dimethylphenol**	930	1.5	RI,MS	Nonanoic acid	1277	1.5	RI,MS
**Benzaldehyde**	960	0.8	RI,MS	2-Undecanone	1292	1.3	RI,MS
**Vinyl amyl ketone**	976	2.7	RI,MS	heptenyl acrolein	1314	1.2	RI,MS
**Octanone**	984	2.6	RI,MS	Undecenal	1362	1.0	RI,MS
**2-Amylfuran**	990	3.1	RI,MS	Capric acid	1372	3.8	RI,MS
**Octanal**	1000	1.1	RI,MS	Benzenepropanoic acid	1415	2.1	RI,MS
**1,8-cineole**	1028	1.2	RI,MS	Hexylresorcinol	1446	1.4	RI,MS
**Octilin**	1070	0.8	RI,MS	Neryl acetone	1451	1.8	RI,MS
**Linalool**	1099	1.5	RI,MS	Lauric acid	1569	4.9	RI,MS
**Nonanal**	1103	6.6	RI,MS	Tetracosamethyl-Cyclododecasiloxane	1696	1.1	RI,MS
**Camphor**	1141	0.6	RI,MS	Pentadecanone	1696	0.6	RI,MS
**Thiophene,2,3-dimethyl**	1145	0.9	RI,MS	Tetradecanoic acid	1765	2.4	RI,MS
**Nonenal**	1158	1.0	RI,MS	Dibutyl phthalate	1958	10.3	RI,MS
**Borneol**	1165	1.8	RI,MS	Palmitic acid	1965	3.7	RI,MS
**1-Nonanol**	1171	2.5	RI,MS	5-Dodecyldihydro furanone	2099	1.4	RI,MS
**Naphthalene**	1179	1.4	RI,MS	Pentacosane	2499	1.3	RI,MS
**2-Decanone**	1192	1.2	RI,MS	Heptacosane	2892	0.9	RI,MS

a: Retention indices using a DB-5 column.

b: Relative area (peak area relative to total peak area).

Nineteen compounds were identified in the oil of the plant representing 88.8% of the oil composition. The main compounds were dibutyl phthalate (10.3%), hexanal (9.5%) and nonanal (6.6%). Other notable constituents were decanal (5.5%), lauric acid (4.9%) and capric acid (3.8%). As can be seen from the above information, the oil was characterized by large amounts of monoterpenes (3.6%), acid compounds (19.4%) and organic compounds (83.7%). Various species of the genus *Trigonella *are important from the point of medical and culinary aspects view. Among these, *T. foenum-graecum *L. is commonly grown as a vegetable. This species has been used as an anthelmintic against the most common nematodes. It has also been used in Indian folk medicine as an antipyretic, diuretic, and supportive, and for treatment of dropsy, heart disease, chronic cough, and spleen and liver enlargement. Another species, *T. caerulea *is used as a food in the form of young seedlings and in cheese making (23-26). The major constituents of *T. disperma *were pentacosane (27.3%), spathulenol (17.3%), and caryophyllene oxide (7.9%) (27). The comparison of chemical composition of the two oils with *T. monantha *shows much similarity, although the plant species belongs to the same genus. Of all the identified components, only spathulenol occurs in different amounts in both species. In a previous investigation, the oil obtained from *T. foenum-graecum *was identified as containing cadinene (27.6%), eudesmol (11.2%), and bisabolol (10.5%) detected (6). The essential oil of *T. monantha*, a widespread species of the genus in the western part of Iran, has not been studied previously. Here for the first time, we report the composition of the essential oils of *T. monantha *which is an endemic species growing wild in Iran.

*Antibacterial activity*

The *in-vitro *antibacterial activity of *T. monantha *seeds and aerial parts against nine bacteria species, selected as representatives of the classes of Gram-positive and Gram-negative and known to cause respiratory, gastrointestinal and skin and urinary disorders, was evaluated by determining the minimum bactericidal concentration, using the broth dilution method. The results obtained in the antibacterial assay are shown in Table 2. Our sample showed activity particularly against the Gram-positive bacteria, as evidenced by the lower MIC values found in Gram-positive bacteria. Gram-negative bacteria appeared not to be sensitive to the oil.

**Table 2:** Antimicrobial activity of ME, HE and CE of *T . monantha*, using agar well diffusion

**Microorganisms**	**Gram+/-**	**EO** **a**	**ME**b		**HE**c		**CE**d	
			**Seeds**	**Aerial parts**	**Seeds**	**Aerial parts**	**Seeds**	**Aerial parts**
*Bacillus antracila *(PTCC 1274)	+	25	5	5	-	5	-	5
*Bacillus cereus *(PTCC 1247)	+	20	-	-	-	-	-	20
*Bacillus subtilis *(PTCC 1023)	+	5	5	-	20	10	20	25
*Escherichia coli *(ATCC 25922)	-	25	-	-	-	10	A little	10
*Pseudomonas sp.*(ATCC 85327)	-	medium	5	5	10	5	A little	-
*Salmonella typhi *(ATCC 1231)	-	10	-	-	-	10	-	10
*Shikla fleksheneri *(ATCC 1042)	-	-	-	-	-	-	-	-
*Staphylococcus epidermaidis *(ATCC 12228)	+	-	-	A little	-	5	-	10
*Staphylococcus aureus * (ATCC 25923)	+	-	20	-	-	10	-	-

Agar disc diffusion method Diameter of inhibition zone (mm) including disk diameter of 100 mm. a=Essential Oil (EO); b=Methanol Extract (ME); c=Hexane Extract (HE); d=Chloroform Extract (CE)

*2.3. Antioxidant activity of extract (ME, CE and HE)*

*Amount of DPPH*

**Figure 1:**
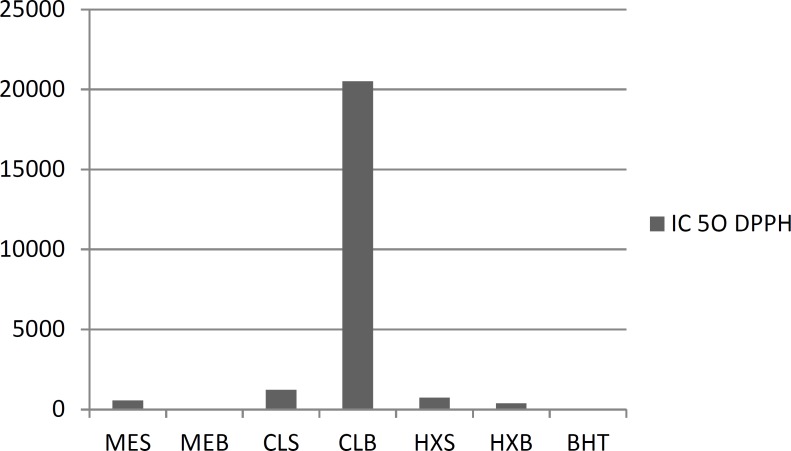
Free radical-scavenging capacities of the aerial parts extract *T. monantha *measured in DPPH assay

As shown in Figure 1, the ME, CE and HE were able to reduce the stable radical, DPPH to the yellow colored diphenylpicrylhydrazine with an IC50 value of methanolic leaves equal to 49.58 ± 0.008 g/mL, followed by the CE of aerial parts 2052.20 ± 0.008 g/mL. In all of the studied samples, the ME of aerial parts had the strongest free radical-scavenging activity. The concentration of the positive control BHT 

required to scavenge 50% of the free radical (IC50) was 18.3 ± 0.006 μg/mL.

*Amount of β-caroten*

**Figure 2:**
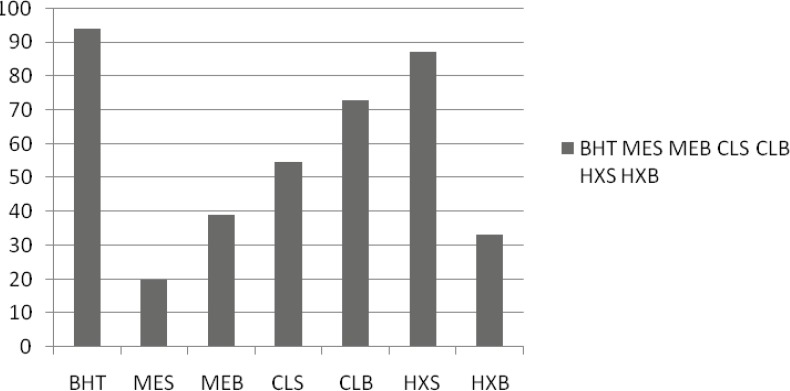
Antioxidant activity of aerial parts extract *T. monantha *defined as inhibition percentage in *β*-carotene-linoleic acid assay

In the case of the linoleic acid system, in general, HE of seeds’ extracts seems to inhibit the oxidation of linoleic acid and that is an important issue in food processing and preservation (Figure 2). Antioxidants minimize the oxidation of lipid components in cell membranes or inhibit the volatile organic compounds and the conjugated diene hydroperoxides arising from linoleic acid oxidation that are known to be carcinogenics. In general, a similar activity pattern to that seen in the first system was observed. Among the extracts prepared with various solvents, the strongest effect was supplied by the HE of seed 87.03.

*Amount of total phenolics*

**Figure 3:**
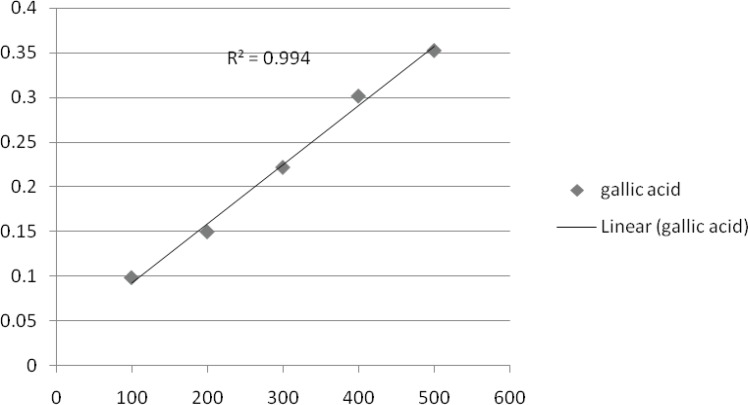
Antioxidant activity of aerial parts extract *T. monantha *defined as the inhibition percentage in total phenolic-gallic acid assay

Based on the absorbance values of the various extract solutions reacted with Folin-Ciocalteu reagent and compared with the standard solutions of gallic acid equivalents as described above, total phenolics are shown in Figure 3. The amount of total phenolics was the highest in the ME of aerial parts extract (22.36 mg galic acid/g). The lowest value was exhibited by the CE of seeds (2.60 mg gallic acid/g). Indeed, when the results given in Figures 1-3 are compared, it is seen that the phenolic content was high in polar extracts. It seems clear that the presence of polar phenolics is fundamental in the evaluation of free radical-scavenging. Besides, the highest activity, seen for the polar sub-fraction of the methanol extract, reflects the radical-scavenging characteristics of these phenolics. The key role of phenolic compounds, as scavengers of free radicals, is emphasised in several reports (28). Moreover, radical-scavenging activity is one of the various mechanisms contributing to the overall activity and thereby, creating the synergistic effects. On the other hand, total antioxidant activities of the non-polar ME, CE and HE could also be attributed to the volatile components, since they are still present in these extracts.

**Figure 4:**
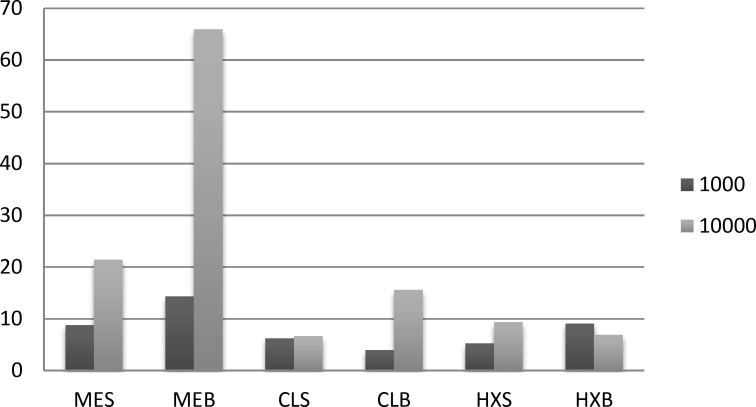
ABTS test for the aerial parts and seeds of *T. monantha*

*Amount of ABTS*

As shown in ABTS, the ME seed extract was able to reduce the stable radical, that was 17.14 μ mol trolox/g in 10000 concentration.

Antioxidant activity of the stem and root oils of *T. monantha *was determined by two different test systems, DPPH and *β*-carotene/linoleic acid. In the DPPH method, the antioxidants react with the stable free radical, *i.e. *DPPH (deep violet color), and convert it to 1,1-diphenyl-2-picrylhydrazine with discoloration. The degree of discoloration indicates the free radical scavenging potentials of the sample/antioxidant; it has been found that known antioxidants such as cysteine, glutathione, ascorbic acid, tocopherol and polyhydroxy aromatic compounds (hydroquinone, pyrogallol, *etc.*) reduce and decolourize the DPPH by their hydrogen-donating ability. In the present study, the weakest radical scavenging activity was occurred with the root oil (722.8 ± 4.3 μg mL−1). On the other hand, none of the samples showed activity as strong as the positive control BHT (18.0 ± 0.4 μg mL−1). Particularly, the synergistic effects of phenolic acids, *e.g. *rosmarinic acid and polyphenols, as well as other chemicals such as flavonoids, could be also taken into account for the radical scavenging activity observed in methanol extracts.

In the *β*-carotene/linoleic acid model system, *β*-carotene undergoes rapid discoloration in the absence of an antioxidant. This is because of the coupled oxidation of *β*-carotene and linoleic acid, which generates free radicals. The linoleic acid free radical formed upon the abstraction of a hydrogen atom from its diallylic methylene group attacks the highly unsaturated *β*-carotene molecules. As a result, *β*-carotene is oxidized and broken down in part; subsequently, the system loses its chromophore and characteristic orange color, which is spectrophotometrically monitored. The %inhibition capacity of the stems oil (25.3 ± 1.2) was found to be superior to the sample, which was the nearest to the inhibition capacity of the positive control BHT (96.6 ± 0.9) (Table 3). The auto-oxidation of linoleic acid without volatiles and methanol extracts accompanies the rapid increase of peroxides. According to Farag Badei and El-Baroty (29), there is a relationship between the inhibition of hydroperoxide formation and the presence of some phenolic nuclei in essential oils and extracts. The antioxidative effectiveness in natural sources has been reported to be mostly due to the phenolic compounds.

**Table 3:** Antioxidative capacities of the seeds and aerial parts of *T. monantha*

**Seeds**	**IC** **50** **, μg/mL**	**Aerial parts**	**IC** **50** **, μg/mL**
HEb (10000ppm)	740.4	HE (10000ppm)	390.3
CEc (10000ppm)	1230.2	CE (10000ppm)	20520.2
MEd (1000ppm)	566.3	ME (1000ppm)	49.6

Results are means of three different experiments. bMethanol Extract (ME); cChloroform Extract (CE); dHexane Extract (HE)

*Antimicrobial activity*

The essential oil of *Pseudomonas *was found to possess medium activity against the *Bacillus antracila *and *Bacillus cereus, Bacillus subtilis, Escherichia coli *and *Salmonella typhi, *and had broader activity against the tested microorganisms and did not exhibit activity, including *Shikla fleksheneri, Staphylococcus epidermidis *and *Staphylococcus aureus. *On the other hand, no activity was exhibited by either of the plant extracts (Table 2). When compared to the extracts, the essential oils showed stronger and broader activity. In the comparison of ME, CE and HE, seeds of ME, CE burk and HE were stronger than the others. As a result of these findings, antimicrobial activities of essential oils could be attributed to camphor, derivatives and borneol. The synergistic effects of these chemicals with each other and minor constituents of the essential oil should be taken into consideration for the activity. The mechanism of terpenes action is not fully understood but it is thought to involve the membrane disruption by the lipophilic compounds (18).

## Conclusions

In the present assay, we studied the content and representation of particular antioxidant and antibacterial activities of various extracts and chemical composition of the essential oil of *Trigonella monantha *C. A. Mey. subsp. *monantha *by gas chromatography (GC) and gas chromatography-mass spectroscopy (GC-MS), from the compounds derived from the aerial parts. We identified 19 major and minor constituents of essential oils from selected species. In oil from the dibutyl phthalate (10.3%), hexanal (9.5%) and nonanal (6.6%) were the major components in aerial parts. These constituents represented 88.8% of the total oil. Organic compounds represented the most abundant constituents of the oil (83.7%). *T. monantha *oils showed antibacterial activity, particularly towards Gram-positive bacteria. Extract the strongest activity was exhibited of the *T. monantha *of ME for aerial parts was determined to be 49.58 μg/mL, the strongest activity was exhibited by ME. Data up to the largest near BHT amount was 18.3 ± 0.3 μg/mL. In the *β*-carotene linoleic acid system, *T. monantha *HE of seed exhibited 87.03 and CE of leaves exhibited 72.97 inhibitions against the linoleic acid oxidation. In the ABTS, *T. monantha *ME of seed exhibited 17.14 μmol trolox/g (TEAK) that was the strongest activity among the other samples. 
